# Complex structure of type VI peptidoglycan muramidase effector and a cognate immunity protein

**DOI:** 10.1107/S090744491301576X

**Published:** 2013-09-20

**Authors:** Tianyu Wang, Jinjing Ding, Ying Zhang, Da-Cheng Wang, Wei Liu

**Affiliations:** a National Laboratory of Biomacromolecules, Institute of Biophysics, Chinese Academy of Sciences, Beijing 100101, People’s Republic of China; b University of Chinese Academy of Sciences, Beijing 100049, People’s Republic of China; c Institute of Immunology, The Third Military Medical University, Chongqing 400038, People’s Republic of China

**Keywords:** muramidases, peptidoglycan, effectors, immunity, calcium binding, interaction

## Abstract

The type VI secretion system (T6SS) is a bacterial protein-export machine that is capable of delivering virulence effectors between Gram-negative bacteria. The T6SS of *Pseudomonas aeruginosa* transports two lytic enzymes, Tse1 and Tse3, to degrade cell-wall peptidoglycan in the periplasm of rival bacteria that are competing for niches *via* amidase and muramidase activities, respectively. Two cognate immunity proteins, Tsi1 and Tsi3, are produced by the bacterium to inactivate the two antibacterial effectors, thereby protecting its siblings from self-intoxication. Recently, Tse1–Tsi1 has been structurally characterized. Here, the structure of the Tse3–Tsi3 complex is reported at 1.9 Å resolution. The results reveal that Tse3 contains a C-terminal catalytic domain that adopts a soluble lytic transglycosylase (SLT) fold in which three calcium-binding sites were surprisingly observed close to the catalytic Glu residue. The electrostatic properties of the substrate-binding groove are also distinctive from those of known structures with a similar fold. All of these features imply that a unique catalytic mechanism is utilized by Tse3 in cleaving glycosidic bonds. Tsi3 comprises a single domain showing a β-sandwich architecture that is reminiscent of the immunoglobulin fold. Three loops of Tsi3 insert deeply into the groove of Tse3 and completely occlude its active site, which forms the structural basis of Tse3 inactivation. This work is the first crystallographic report describing the three-dimensional structure of the Tse3–Tsi3 effector–immunity pair.

## Abstract 2

The structure of the Tse3–Tsi3 complex associated with the bacterial type VI secretion system of *P. aeruginosa* has been solved and refined at 1.9 Å resolution. The structural basis of the recognition of the muramidase effector and its inactivation by its cognate immunity protein is revealed.

## 1. Introduction

The structural integrity of the bacterial cell is largely determined by its cell wall. Without a rigid intact cell wall, bacteria are not able to maintain cell shape, resist the internal osmotic pressure or prevent lysis (Koch, 1988 ▶). In Gram-negative bacteria peptidoglycan serves as the major structural component of the cell wall, which forms a meshwork in the periplasm surrounding the entire cell. Peptidoglycan is a single-molecular biopolymer consisting of linear oligomeric glycan strands of alternating *N*-acetylmuramic acid (MurNAc) and *N*-acetylglucosamine (GlcNAc) residues cross-linked by short peptide chains of alternating l-amino and d-amino acids. Owing to its invariant structure and essential roles, the bacterial cell wall often becomes a vulnerable target for lytic enzymes, *e.g.* lysozyme (EC 3.2.1.17; also referred to as muramidase), effective defence molecules that exist in plants or animals to provide innate immunity against bacteria (Bugg *et al.*, 2011 ▶; Hartl *et al.*, 2012 ▶; Saurabh & Sahoo, 2008 ▶).

Besides the immense attacking pressure from eukaryotes, bacteria also face widespread, fierce and deliberate competition for limited resources from other microbes (Hibbing *et al.*, 2010 ▶; Brogden *et al.*, 2005 ▶), which has shaped the evolution of a number of antagonistic pathways which commonly target bacterial cell-wall peptidoglycan (Hayes *et al.*, 2010 ▶; Konovalova & Søgaard-Andersen, 2011 ▶; Rendueles & Ghigo, 2012 ▶). Specific mechanisms are required to allow cell-wall-targeting molecules to penetrate the outer membrane and to access peptidoglycan.

Many bacteria use specialized secretion systems to inject virulence factors into the target host cells. The type VI secretion system (T6SS) was recognized as a distinct class of bacterial protein secretion apparatus in 2006 (Pukatzki *et al.*, 2006 ▶; Mougous *et al.*, 2006 ▶). Recent studies, mostly contributed by Mougous and coworkers, have identified T6SS as a complex and widely distributed protein-export machine that is capable of cell-contact-dependent targeting of effector proteins between Gram-negative bacteria (Hood *et al.*, 2010 ▶; Russell *et al.*, 2011 ▶; Schwarz *et al.*, 2010 ▶). Their pioneering work shows that the haemolysin co-regulated protein (Hcp) secretion island I-encoded T6SS of the bacterium *Pseudomonas aeruginosa*, a human pathogen of cystic fibrosis (CF) lung disease (Ratjen, 2009 ▶), targets at least three proteins, termed type VI secretion exported 1–3 (Tse1–Tse3), to the periplasmic space of rival bacteria (Hood *et al.*, 2010 ▶). Among the delivered proteins, Tse1 and Tse3 are biochemically characterized as lytic antibacterial enzymes that degrade the peptidoglycan of recipient bacterial cells, thereby causing rapid cell lysis of competing bacteria in the environment (Russell *et al.*, 2011 ▶). It is now known that Tse1 functions as a peptidoglycan amidase with specificity towards γ-d-glutamyl-*meso*-2,6-diamino­pimelic acid (d-Glu-mDAP), while Tse3 functions as a mur­amidase that hydrolyzes the β-1,4-linkage between MurNAc and GlcNAc. Since the peptidoglycan of *P. aeruginosa* itself is not inherently resistant to Tse1 or Tse3, to protect its siblings the bacterium synthesizes two immunity proteins, Tsi1 and Tsi3, in the periplasmic compartment to specifically bind the cognate virulence effectors delivered from neighbouring cells, thereby resisting self-intoxication (Russell *et al.*, 2011 ▶). These two effector–immunity pairs jointly confer a growth advantage to *P. aeruginosa* over its competitors.

Since the milestone work on the identification of these peptidoglycan-degrading effectors, both Tse1 and Tse3 have attracted intensive research from biochemists, geneticists, bioinformaticians and structural biologists. To date, crystal structures of Tse1 alone and/or in the Tse1–Tsi1 complex have been independently determined by us (Ding *et al.*, 2012 ▶) and in other laboratories (Shang *et al.*, 2012 ▶; Zhang *et al.*, 2012 ▶; Chou *et al.*, 2012 ▶; Benz *et al.*, 2012 ▶), and in-depth mechanical insights into the recognition and cleavage of the murein peptide substrate by Tse1 as well as the structural basis of the Tse1–Tsi1 interaction were gained from analyses of these structures. Studies of Tse3 and Tsi3, however, are relatively incomplete, as the relevant structural information is unfortunately still unavailable. As an enzyme that cleaves the MurNAc–GlcNAc bond in the glycan backbone, Tse3 has been functionally characterized as possessing lysozyme-like activity rather than lytic transglycosylase activity (Russell *et al.*, 2011 ▶). In comparison with Tse1, Tse3 shows an even lower amino-acid sequence homology to known muramidases or lytic trans­glycosylases, despite containing a sequence motif that includes a catalytic glutamic acid found in muramidases (Russell *et al.*, 2011 ▶). For a better understanding of the enzymatic mechanism of Tse3 and the self-protective strategy implemented *via* the recognition and binding of Tse3 by Tsi3, detailed structural information regarding this effector–immunity pair is needed.

In this study, we determined the crystal structure of the Tse3–Tsi3 complex from *P. aeruginosa*, which reveals unprecedented folds for both proteins. The refined structure at 1.9 Å resolution also unexpectedly shows three calcium-binding sites in Tse3, one resembling the EF-hand motif and the other two being non-EF-hand sites close to the catalytic Glu residue. The crystallographic results reported here not only establish a structural framework for understanding the enzymatic mechanism underlying Tse3 lysozyme activity, but also allow us to scrutinize the structural basis for the mutual recognition and interaction between the effector and the cognate immunity protein. Moreover, the structural information presented in this article may be applicable in developing new therapeutic strategies for dealing with human diseases caused by polymicrobial infections.

## 2. Experimental procedures

### 2.1. Expression and purification

Nucleotide sequences encoding full-length Tse3 and truncated Tsi3 without the N-terminal signal peptide (residues 1–­20) were synthesized with proper optimization of the GC content (Sangon Biotech, Shanghai, People’s Republic of China). Both genes were inserted into pET-22b vector (Novagen, Darmstadt, Germany) between the *Nde*I and *Xho*I endonuclease cleavage sites for cytoplasmic expression of recombinant protein with a C-terminal His tag. Protein expression in *Escherichia coli* strain BL21(DE3) was induced with 50 µ*M* isopropyl β-d-1-thiogalactopyranoside when the bacterial cultures reached an OD_600_ of 0.6–0.8. After 24 h of incubation at 289 K, the bacterial cells were harvested by centrifugation at 4000*g* for 30 min.

For purification of Tse3, the cell pellets were resuspended in lysis buffer [150 m*M* NaCl, 20 m*M* HEPES pH 7.5, 10 m*M* imidazole, 5 m*M* β-mercaptoethanol, 5%(*v*/*v*) glycerol] with the addition of 0.1 m*M* PMSF after discarding the supernatant. Bacteria were lysed by sonication on ice at 200 W using 3 s pulses with 7 s intervals for 16.5 min before the removal of insoluble debris by centrifugation for 30 min at 16 000*g* and 277 K. The recombinant protein was initially purified using Ni^2+^–NTA His·Bind resin (Novagen, Darmstadt, Germany) and was immediately diluted with buffer *A* [50 m*M* NaCl, 20 m*M* HEPES pH 7.5, 1 m*M* dithiothreitol (DTT), 5%(*v*/*v*) glycerol]. The eluted fraction was further purified by cation-exchange (using a 5 ml HiTrap SP HP column, GE Healthcare, Uppsala, Sweden) and size-exclusion (using a HiLoad 16/60 Superdex 75 column, GE Healthcare) chromatography. Selenomethionine-substituted (SeMet) Tse3 was produced in the methionine-auxotrophic *E. coli* strain B834 (DE3). An identical protocol to that used for the native protein was applied for the purification of the SeMet derivative, but with 10 m*M* β-­mercaptoethanol added to the lysis buffer and 5 m*M* DTT added to buffer *A*. Native Tse3 was stored in 150 m*M* NaCl, 20 m*M* HEPES pH 7.5, 1 m*M* DTT, 5% glycerol after purification and the SeMet protein was stored in the same buffer but containing 4 m*M* DTT and 0.2 m*M* EDTA.

For purification of Tsi3, the bacterial pellets were resuspended in the same lysis buffer but without glycerol after cell harvesting. Tsi3 was purified using the same three-step procedure as Tse3 and was stored in 150 m*M* NaCl, 20 m*M* Tris–HCl pH 7.5, 1 m*M* DTT. The Tse3–Tsi3 complex was obtained by mixing the two proteins with excess Tsi3 to saturate the Tse3 and was further purified by size-exclusion chromatography using a HiLoad 16/60 Superdex 75 column (GE Healthcare, Uppsala, Sweden). The purified complex protein was stored in the same buffer as used for Tsi3 and was frozen at 193 K until further use.

### 2.2. Crystallization and data collection

The Tse3–Tsi3 complex was concentrated to approximately 5 mg ml^−1^ prior to crystallization trials, which were carried out at 293 K using the hanging-drop vapour-diffusion method. The drop in each well was formed by mixing 1 µl protein solution with 1 µl screen solution and was equilibrated against 0.5 ml reservoir solution. An initial crystallization condition was established using the Index kit from Hampton Research (Aliso Viejo, California, USA). After thorough optimization, X-ray diffraction-quality crystals of SeMet protein were grown in 25%(*v*/*v*) Jeffamine ED-2001, 0.1 *M* bis-tris pH 6.5.

All crystals used for X-ray data collection were transferred into cryoprotectant [reservoir solution supplemented with 10%(*v*/*v*) ethylene glycol] for approximately 10 s before being mounted in nylon cryoloops (Hampton Research) and flash-cooled in a stream of liquid nitrogen for optimal cryoprotection. A single-wavelength anomalous diffraction (SAD) data set was collected for the SeMet-labelled Tse3–Tsi3 complex at 100 K at a wavelength of 0.9795 Å on beamline 17A of the Photon Factory, KEK, Japan using an ADSC Quantum 315r CCD detector. All X-ray diffraction data were indexed, integrated and scaled using *iMosflm* (Battye *et al.*, 2011 ▶) and *SCALA* from the *CCP*4 program suite (Winn *et al.*, 2011 ▶).

### 2.3. Structure determination and refinement

The Tse3–Tsi3 complex structure was determined by the SAD technique. Phase calculation and density modification were performed using the *PHENIX* program suite (Adams *et al.*, 2010 ▶), after which a partial model was automatically built. The rest of the model was manually completed using the *Coot* graphics package (Emsley *et al.*, 2010 ▶). The structure was refined using *phenix.refine* (Afonine *et al.*, 2012 ▶; Headd *et al.*, 2012 ▶) with manual modelling between refinement cycles. The final model was validated using the *MolProbity* server (Chen *et al.*, 2010 ▶) and was deposited in the Protein Data Bank (PDB) as entry 3wa5. The statistics of data collection, SAD phasing and structure refinement are given in Table 1 ▶.

### 2.4. Metal-ion detection

Three divalent metal-binding sites were clearly observed on the protein surface of Tse3, as indicated by strong spherical electron density in the *F*_obs_ − *F*_calc_ map at 3.5σ. To identify the correct metal ions that should be modelled in this structure, the elemental composition was determined using inductively coupled plasma atomic emission spectroscopy (ICP-AES). For this experiment, Tse3 alone and the Tse3–Tsi3 complex were produced separately according to the abovementioned protocol and then diluted to concentrations of 0.15 and 0.2 mg ml^−1^, respectively. The spectra of Mg^2+^, Ca^2+^, Co^2+^, Ni^2+^ and Zn^2+^ for the two protein samples were obtained using Vista-MPX ICP-AES (Varian, Palo Alto, California, USA), from which corresponding ion concentrations were measured.

### 2.5. Structure analysis

The final model was sent to the *ProFunc* server (Laskowski *et al.*, 2005 ▶) for topological analysis, the *PIC* server (Tina *et al.*, 2007 ▶) for the analysis of protein–protein interfaces and the *DALI* server (Holm & Rosenström, 2010 ▶) for a structural neighbour search. Structural comparison and structure-based sequence alignment were performed using the *DaliLite* server (http://www.ebi.ac.uk/Tools/structure/dalilite/). All figures representing the Tse3–Tsi3 complex structure were prepared using the *PyMOL* molecular-visualization program (v.1.3r1; Schrödinger).

### 2.6. Mutational study and Tse3–Tsi3 kinetic assay

In order to investigate the structural determinants of Tse3–­Tsi3 recognition and binding, a series of Tsi3 mutants including R60A, S99A, E103A, Q124A and E126A were produced by site-directed mutagenesis. These mutants together with the wild-type protein were subjected to a kinetics assay at 298 K using a Biacore 3000 surface plasmon resonance (SPR) instrument (GE Healthcare, Uppsala, Sweden). A running buffer consisting of 50 m*M* HEPES pH 7.5, 100 m*M* NaCl, 0.005%(*v*/*v*) Tween-20 was prepared, vacuum-filtered and degassed immediately prior to the experiment. The Tse3 protein dissolved in 10 m*M* sodium acetate pH 5.0 at a concentration of 15 µg ml^−1^ was immobilized on six flow cells of a CM5 sensor chip with densities from 2689.1 to 3396.3 response units. The wild-type and mutant Tsi3 proteins were transferred to 150 m*M* NaCl, 20 m*M* HEPES pH 7.5 using three cascaded 5 ml HiTrap desalting columns before sample injection.

Kinetic profiling was performed using the single-cycle kinetics method (Tang *et al.*, 2006 ▶). In each analysis cycle, increasing sample concentrations were injected consecutively over the Tse3 surfaces and a reference blank flow cell at a flow rate of 30 µl min^−1^. Tsi3 was diluted in the running buffer to five different concentrations in the range 0.02–1.62 µ*M* for the wild-type protein and the E103A and Q124A mutants and in the range 0.1–8.1 µ*M* for the R60A, S99A and E126A mutants. Protein samples were injected for 60 s at each concentration. Protein dissociation was allowed by running the buffer alone for 10 min after the last injection. The resultant data were analyzed using the *Biacore 3000* evaluation software by fitting to a 1:1 Langmuir binding model.

## 3. Results

### 3.1. Overall structure

**Figure 1:**
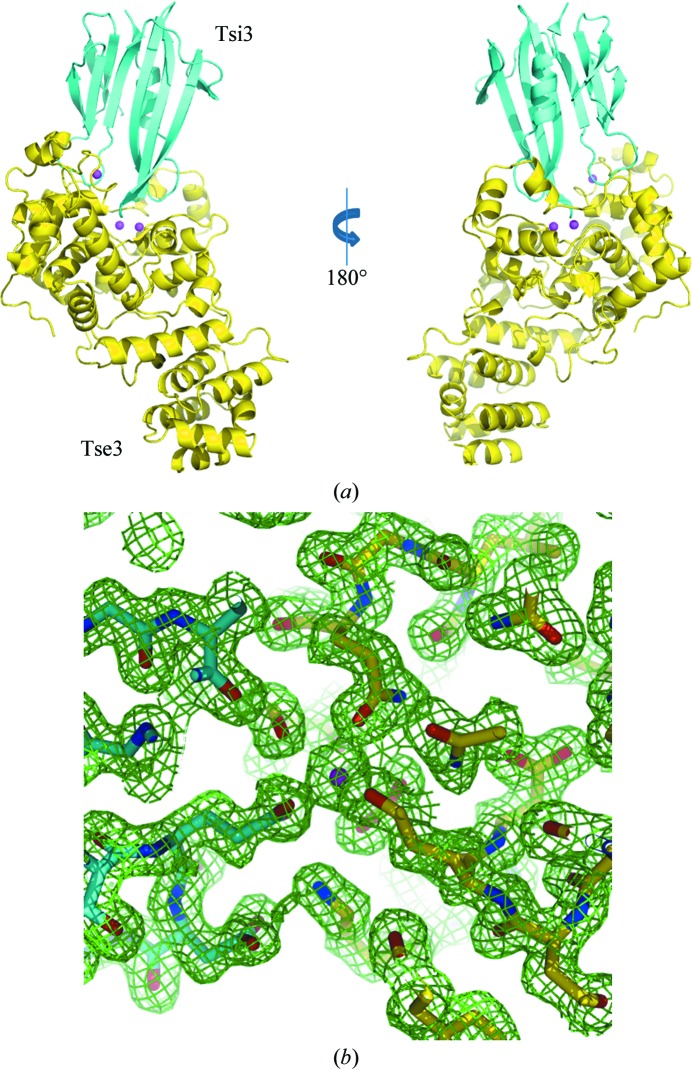
The overall structure and model quality of the Tse3–Tsi3 complex. (*a*) Ribbon diagram showing the binary complex, in which Tse3 is coloured yellow and Tsi3 cyan. The Ca^2+^ ions present in Tse3 are represented by magenta balls. (*b*) Representative 2*F*_obs_ − *F*_calc_ density map contoured at 1.0σ of the interface between Tse3 and Tsi3. The protein stick model is coloured by element: carbon, yellow (Tse3) or cyan (Tsi3); oxygen, red; nitrogen, blue.

The crystal structure of the Tse3–Tsi3 complex was solved by SAD phasing using selenomethionine-substituted protein and was refined at 1.9 Å resolution. The protein crystallized in space group *P*4_3_2_1_2 with a single Tse3 molecule and a single Tsi3 molecule in the asymmetric unit (Fig. 1 ▶ *a*). The two protein molecules form a compact complex with overall dimensions of 60 × 58 × 93 Å, and the molar ratio of Tse3 and Tsi3 agrees well with the 1:1 stoichiometry observed in solution. The final model comprises 524 amino-acid residues (400 in Tse3 and 124 in Tsi3) and 560 heterologous atoms including three metal ions and 525 solvent molecules. Almost all protein residues are well defined in the electron-density map (Fig. 1 ▶ *b*), except for residues 13–19 of Tse3, which did not show interpretable density in either the 2*F*_obs_ − *F*_calc_ or the *F*_obs_ − *F*_calc_ maps and had to be omitted from the model. The quality of the refined complex model appeared to be good in terms of both crystallographic and stereochemical parameters, which are given in Table 1 ▶.

### 3.2. The structure of Tse3

The Tse3 molecule displays a tilted Y-shape in the complex. In the orientation shown in Fig. 1 ▶(*a*), two lobes stretch from a long groove in which Tsi3 sits on Tse3. The secondary structure of Tse3 is dominated by α-helices, with 59% of all observed amino-acid residues distributed in a total of 28 helices, including six 3_10_-helices (Supplementary Fig. S1*a* 1). In sharp contrast, only one small β-sheet comprising two short antiparallel strands, β1 (residues 274–276) and β2 (residues 281–282), exists in the model. The α-helices are packed tightly, which results in extensive helix–helix contacts that contribute greatly to structural integrity and stability.

**Figure 2:**
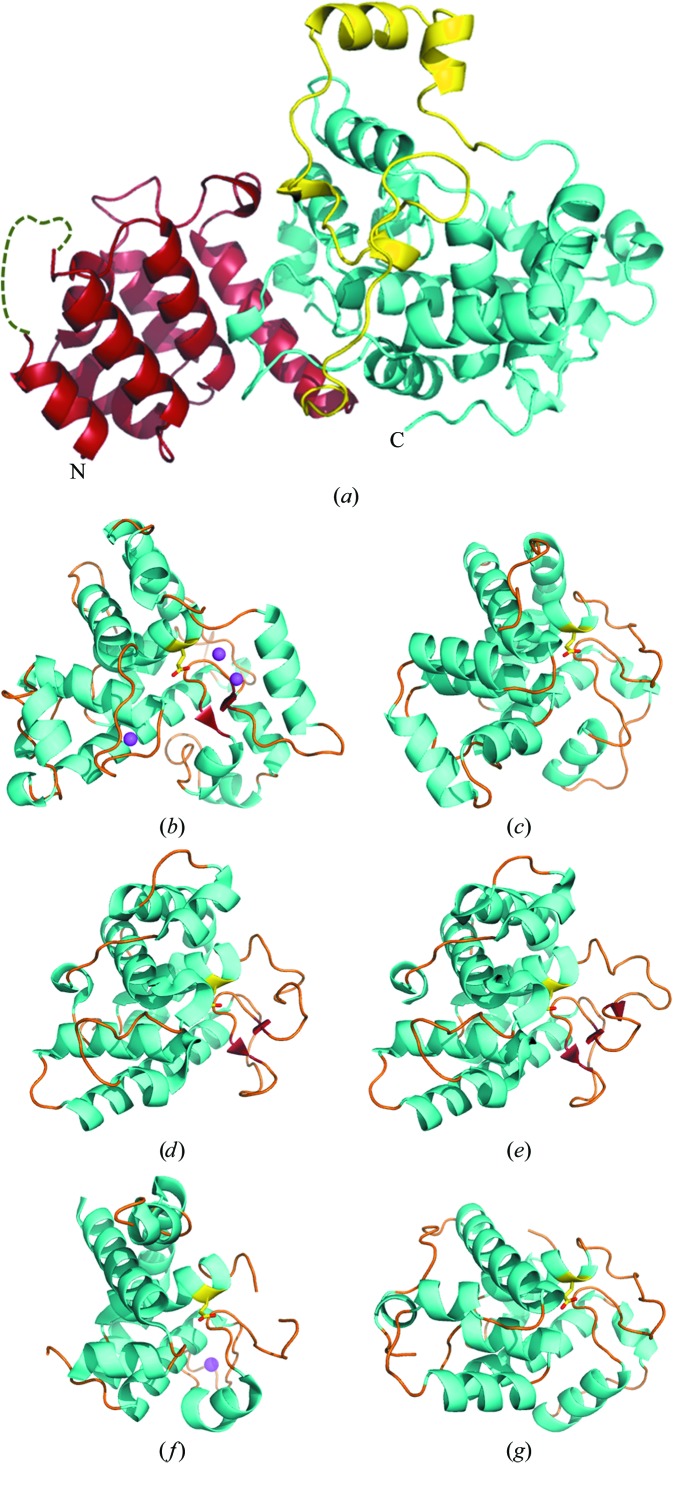
The structure of Tse3 and its catalytic domain in comparison with representative structures containing the SLT domain (PF01464 in the Pfam database). (*a*) Ribbon model of the overall structure of Tse3. The N-terminal domain and the C-terminal catalytic domain are coloured red and cyan, respectively, while the linker segment is shown in yellow. The loop that is not visible in the electron-density map (residues 13–19) is schematically represented by a green dashed curve. (*b*) Ribbon diagram of the C-terminal SLT-like domain of Tse3. (*c*–*g*) Representative SLT domains of the lytic transglycosylases MltE (PDB entry 2y8p) (*c*), Slt35 (PDB entry 1qus) (*f*) and Slt70 (PDB entry 1qsa) (*g*) from *E. coli* and the goose-type lyzosymes from goose egg (PDB entry 153l) (*d*) and Atlantic cod (PDB entry 3gxr) (*e*). Catalytic glutamic acids and metal ions are highlighted as stick models and magenta spheres, respectively.

Given its compact fold, it is not easy to make out domain boundaries in this model merely by eye. A structural neighbour search performed using the *DALI* server (Holm & Rosenström, 2010 ▶) suggests that the C-terminal peptide region from residues 183 to 408 resembles the lysozyme-like fold (SCOP 53954). The domain-organization prediction server *pDomains* (Veretnik *et al.*, 2004 ▶) also recognized this peptide segment as an independent structural unit similar to the soluble lytic transglycosylase (SLT) family (Pfam PF01464). Based on these analyses, we considered decomposing the Tse3 structure into three structural modules, all of which are sequentially consecutive and make good contact with each other. Globular domains at each terminus were defined as the peptide regions from residues 2 to 126 and 183 to 408, with a linker segment in between (Fig. 2 ▶ *a*).

Six helices (α1, α2, α3, α5, α6 and α8) constitute an antiparallel helical bundle in the N-terminal domain (NTD), while α5 and α7 are oriented perpendicular to the bundle. Several β-­turns but no β-strands are present in this domain. No structural neighbours of this domain were found by the *DALI* server (Holm & Rosenström, 2010 ▶), indicating that the NTD of Tse3 represents a novel fold. The linker segment (residues 127–182) is composed of several flexible loops separated by short helices (α9–12). Most of the amino acids in this module are located on the protein surface and hence show a higher degree of freedom. Two helices, α11 and α12, are involved in the formation of one lobe on the top of Tse3.

### 3.3. The catalytic domain of Tse3

The C-terminal domain is the catalytic domain of Tse3 as it contains Glu250, the catalytic glutamic acid identified by Russell *et al.* (2011 ▶). This module covers 56% of the amino acids in the primary sequence and forms the upper part of the Y-shaped molecule in the orientation shown in Fig. 1 ▶(*a*). It is made up of 16 α-helices from α13 to α28 and the single β-sheet present in the whole protein (Fig. 2 ▶ *b*). A long groove is formed on top of α17 and the small β-sheet and is flanked by two lobes, one involving α13 and α18 and the other involving α27 and α28. As mentioned above, such an α+β structure resembles the lysozyme-like fold present in SLT domains.

The *DALI* server was used to search for structural neighbours of Tse3. All proteins with a *Z*-score of ≥8.0 are either goose-type lysozymes or lytic transglycosylases. Representative structures of those proteins are shown in Figs. 2 ▶(*c*)–2 ▶(*g*), including the lytic transglycosylases MltE (PDB entry 2y8p; Artola-Recolons *et al.*, 2011 ▶), Slt35 (PDB entry 1qus; van Asselt, Dijkstra *et al.*, 1999 ▶) and Slt70 (PDB entry 1qsa; van Asselt, Thunnissen *et al.*, 1999 ▶) from *E. coli* and the goose-type lysozymes from goose egg (PDB entry 153l; Weaver *et al.*, 1995 ▶) and Atlantic cod (PDB entry 3gxr; Helland *et al.*, 2009 ▶). All of these proteins bind peptidoglycan in a long groove containing at least six subsites, −4 to +2, for substrate binding; the cleavage of the β-(1,4)-linkage occurs between subsites −1 and +1. These structures share low sequence identity (11–22%) with Tse3, but all of them can be superimposed on the catalytic domain of Tse3 with an r.m.s.d. in the range 2.7–3.2 Å on 137–173 C^α^ positions. Compared with its structural neighbours, Tse3 displays two distinctive features: (i) it has a larger catalytic domain owing to several helix and loop insertions in comparison with other SLT domains and (ii) it contains three metal-binding sites on the protein surface (Figs. 2 ▶ *b*–2 ▶ *g*).

### 3.4. Calcium-binding sites

Calcium is a biologically important metal that commonly occurs in various eukaryotic metalloproteins, but is also observed in a few prokaryotic proteins (Yáñez *et al.*, 2012 ▶). An example is *E. coli* lytic transglycosylase Slt35, which contains a noncanonical EF-hand Ca^2+^-binding site (van Asselt, Dijkstra *et al.*, 1999 ▶). In this study, three metal-binding sites are surprisingly observed in the Tse3 structure and all of them are exposed on the surface of the catalytic domain (Fig. 2 ▶ *b*). The spherical *F*_obs_ − *F*_calc_ electron density at 3.5σ corresponding to these sites is much stronger than that for water molecules. The sites are all coordinated by 6–7 oxygen ligands from either protein or solvent, which is a strong indicator of Ca^2+^ binding (Kirberger *et al.*, 2008 ▶; Bindreither & Lackner, 2009 ▶). To confirm this, ICP-AES was performed. The measured ion concentrations for Ca^2+^ were 0.34 and 0.42 mg l^−1^ for protein solutions containing Tse3 alone and the Tse3–Tsi3 complex, respectively, which are higher than those of Mg^2+^, Co^2+^, Ni^2+^ and Zn^2+^ by one or two orders of magnitude (Table 2 ▶). The Ca^2+^ concentrations correspond to metal:protein molar ratios of 2.6:1 for Tse3 alone and 3.2:1 for the Tse3–Tsi3 complex, suggesting full occupancy of Ca^2+^ in the metal-binding sites, although no calcium salts were deliberately added to the bacterial growth medium, the buffers used in protein purification or the reservoir solutions used for crystallization. The correctness of Ca^2+^ modelling was indicated by perfect density fitting, comparable *B* factors with those of protein atoms and a reasonable decrease in *R*_work_/*R*_free_ after ten cycles of restrained refinement.

**Figure 3:**
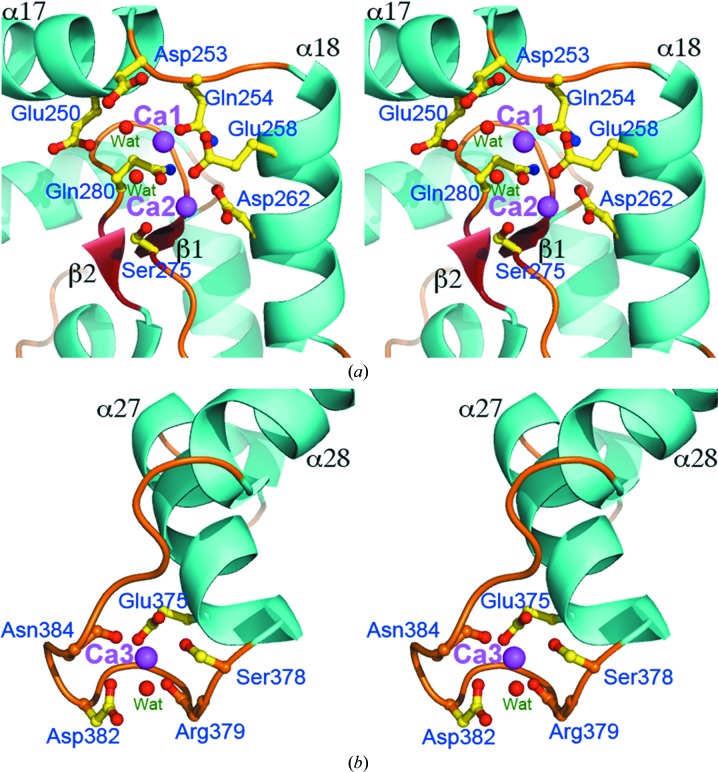
Stereoviews of the three Ca^2+^-binding sites in the structure of Tse3. (*a*) The adjacent non-EF-hand binding sites for Ca1 and Ca2 at a position surrounded by two helices (α17 and α18) and a two-stranded β-sheet. (*b*) The EF-hand binding site for Ca3 coordinated by amino acids on a loop that connects α27 and α28. Ca^2+^ ions are represented by magenta spheres and the ligated amino acids are shown as stick models.

All three Ca^2+^-binding sites are coordinated by oxygen ligands including side-chain carboxyl groups (Asp and Glu), carboxamide groups (Asn and Gln) and hydroxyl groups (Ser), as well as main-chain carbonyl O atoms and waters, with Ca—O distances from 2.4 to 2.7 Å in an overall geometry configured as pentagonal pyramidal (Fig. 3 ▶, Supplementary Fig. S2 and Supplementary Table S1). Two Ca^2+^ ions, named Ca1 and Ca2, are adjacently bound at non-EF-hand binding sites located midway in the groove (Fig. 2 ▶ *b*). The protein ligands involved in these sites are located on discontinuous sequence segments, mostly from the catalytic domain, including Asp253, Gln254, Glu258 and Asp262 in the region from helix α17 to α18, and Ser275 and Gln280 in the single β-sheet (Fig. 3 ▶ *a* and Supplementary Figs. S2*a* and S2*b*). In addition, the side chain of Asn181 from the linker segment is a ligand of Ca1. More strikingly, Glu126 of Tsi3 is involved in Ca2 binding, which may explain the higher occurrence of Ca^2+^ in the solution of the Tse3–Tsi3 complex than in that of Tse3 alone. Although Glu250 does not directly form a Ca—O bond, it is found in the coordination shell and mediates a solvent molecule in Ca1 ligation. Another notable residue is Gln280, the side chain of which directly ligates Ca2 and the main-chain carbonyl O atom of which directly coordinates Ca1 *via* a water molecule (Fig. 3 ▶ *a* and Supplementary Fig. S2*a* and S2*b*). The involvement of these two key catalytic residues in Ca^2+^ binding is astonishing and impressive, as calcium-binding sites so close to the active centre have never been observed in previously characterized lysozymes and lytic transglycosylases. Owing to this unique structural feature, we reason that Tse3 is very likely to exploit an unprecedented mechanism to cleave peptido­glycan substrates in which the calcium ions are involved.

The third Ca^2+^ ion, Ca3, is ligated by the side chains of Glu375, Ser378 and Asp382, as well as the main-chain O atoms of Arg379 and Asn384 (Fig. 3 ▶ *b* and Supplementary Fig. S2*c*). All of these coordinating residues are located on the loop that connects helix α27 to α28. The polypeptide chain contributing them has a conformation that resembles the EF-hand Ca^2+^-binding motif characterized by a helix–loop–helix structure. In contrast to the EF-hand binding site observed in Slt35, in which the central loop consists of 15 amino acids, Ca3 binds in a 12-residue loop in this structure which is consistent with the canonical EF-hand (Kirberger *et al.*, 2008 ▶; Bindreither & Lackner, 2009 ▶; Maki *et al.*, 2011 ▶). Apart from the ligating residues in Tse3, Glu103 of Tsi3 is also involved in Ca3 coordination *via* a solvent molecule. Despite its distance of 16 Å from the catalytic residue, this binding site is positioned close to one end of the substrate-binding groove (Fig. 2 ▶ *b*), implying that Ca3 may also affect catalysis by probably being involved in substrate binding.

### 3.5. Potential substrate-binding sites

**Figure 4:**
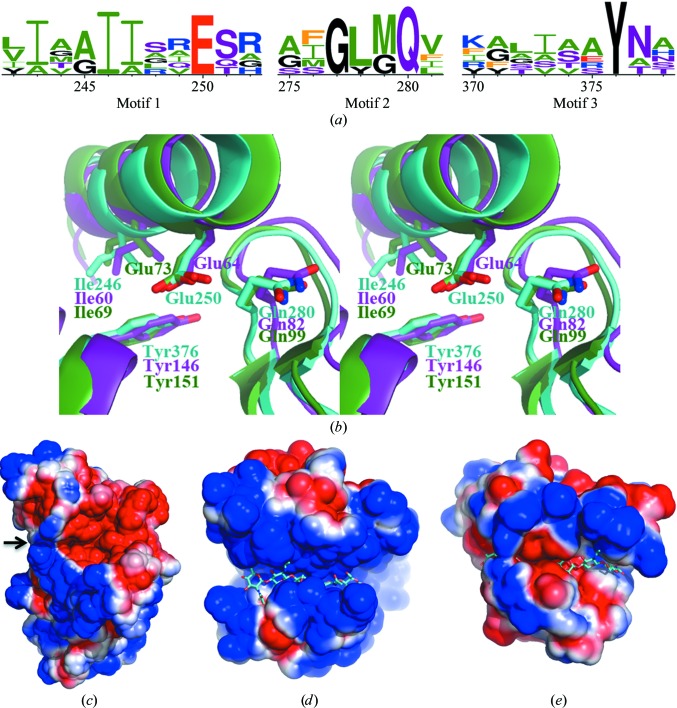
Sequential and structural properties of the potential active site of Tse3. (*a*) Three conserved motifs identified from structure-based sequence alignment between Tse3 and other muramidases containing an SLT domain. Amino-acid conservation is depicted using *WebLogo* (Crooks *et al.*, 2004 ▶). (*b*) Stereoview of structural superimposition of MltE (PDB entry 2y8p; purple) and gLYS (PDB entry 3gxr; green) onto Tse3 (cyan). The invariant residues are highlighted as stick models with element colour indices. (*c*)–(*e*) Electrostatic potential maps calculated on the surface of Tse3 including the three calcium ions (*c*), MltE (*d*) and gLYS (*e*). Positive and negative charges are shown in blue and red colours, respectively. The substrate-binding groove in Tse3 is indicated by a black arrow, while those in the other structures are indicated by bound glycan.

Russell and coworkers identified Glu250 as the catalytic glutamic acid by site-directed mutagenesis (Russell *et al.*, 2011 ▶). They also found two conserved sequential motifs in Tse3 using structure-prediction algorithms, which are separated by 25 amino acids. In this study, structure determination of Tse3 allows us to perform further analyses on the basis of its structural comparison with other structurally characterized peptido­glycan-degrading enzymes con­taining an SLT domain. Indeed, one extra conserved motif close to the C-­terminal end was identified using secondary-structure match­ing (*SSM*) algorithms. Five positions in these three motifs are occupied by invariant amino acids, while the other positions display varying conservation among aligned sequences. The highly conserved residues are Ile246 and Glu250 in motif 1, Gly276 and Gln280 in motif 2 and Tyr376 in motif 3 (Fig. 4 ▶ *a*).

In the structure of Tse3, Ile246 is found in α17, Glu250 is in the loop connecting α17 and α18, and Gln280 is present in the β-hairpin bridging the two β-strands, which forms the only β-­sheet in Tse3. All three residues are located at the bottom of the groove in which glycan substrates are supposed to bind. Gly276 also appears in the β-hairpin, where an amino acid without a side chain is required to form a tight turn. Tyr376 is positioned in α27 flanking the potential peptidoglycan-binding site. Structure superimposition shows that all of these highly conserved residues adopt identical spatial positions with almost the same side-chain rotamers (Fig. 4 ▶ *b*), indicating their indispensable roles in enzyme activity. Apart from Glu250, which is likely to function as the catalytic acid/base in the glycosidic bond-cleavage reaction, Ile246, Gln280 and Tyr376 align the groove that accommodates substrates. The counterparts of Ile246 and Tyr376 in the *E. coli* lytic translycosylase MltE (PDB entry 4hjz; Fibriansah *et al.*, 2012 ▶) and the goose-type lysozyme from Atlantic cod (gLYS; PDB entry 3gxr; Helland *et al.*, 2009 ▶) form hydrophobic interactions with the sugar ring at glycan-binding subsite −2 from opposite sides, and the side chain of Gln82, the counterpart of Gln280 in MltE, forms hydrogen bonds to the *N*-acetyl and C3 hydroxyl groups on GlcNAc at subsite +1 (Fibriansah *et al.*, 2012 ▶). All of these crystallographic data suggest that Ile246, Tyr376 and Gln280 are very likely to serve as substrate stabilizers during enzyme turnover.

The electrostatic potential map calculated on the protein surface reveals that the substrate-binding groove of Tse3 is rich in negatively charged residues (Fig. 4 ▶ *c*), which is another feature that is inconsistent with previously reported SLT domains that adopt a similar fold, *e.g.* the two structures mentioned above. In contrast to MltE (Fig. 4 ▶ *d*) and gLYS (Fig. 4 ▶ *e*), the grooves of which contain more positive charges than negative charges, Tse3 is dominated by acidic residues in its groove. Apparently, this unprecedented charge distribution in Tse3 is attributable to the abundance of glutamic and aspartic acids that coordinate the three Ca^2+^ ions. In other words, the occurrence of these metal ions effectively alters the electrostatic state of the catalytic groove; consequently, the substrate-binding mode in Tse3 probably differs from its structural neighbours.

### 3.6. The structure of Tsi3

The cognate immunity protein Tsi3 sits on top of Tse3 in the orientation shown in Fig. 1 ▶(*a*). It adopts an α+β architecture, differing from the all-β fold of Tsi1 (Ding *et al.*, 2012 ▶) and the all-α fold of Tsi2 (Li *et al.*, 2012 ▶; Wang *et al.*, 2012 ▶). Even so, Tsi3 is built up mostly of β-strands. A total of 73 amino acids (59%) are found in ten β-strands that form two antiparallel β-­sheets, while only nine residues (7%) form a single α-helix. Sheet *A* is constituted by three strands, β1, β2 and β10, and sheet *B* is composed of the other seven strands, β3–β9. The structure comprises two layers, one formed by sheet *A* and the α-helix, and the other formed by sheet *B*. Such a β-sandwich fold can be thought of as a variant version of the jelly-roll topology with an α-helix insertion between the two sheets.

**Figure 5:**
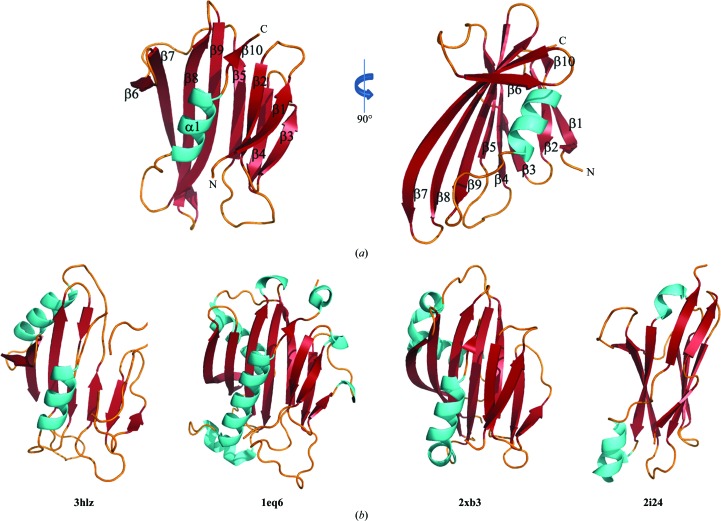
The structure of Tsi3 and comparison with structural neighbours obtained from a *DALI* search (Holm & Rosenström, 2010 ▶). (*a*) Ribbon representation of the structure of Tsi3; (*b*) ribbon models of structures adopting a similar β-sandwich fold. The corresponding PDB codes are given below the structural representations. Loops, α-helices and β-strands are coloured orange, cyan and red, respectively.

*DALI* searches with Tsi3 as the input structure found that the N-terminal domain of an uncharacterized protein from *Bacteroides thetaiotaomicron* (PDB entry 3hlz; Joint Center for Structural Genomics, unpublished work) showed the highest structural similarity to Tsi3 (r.m.s.d. of 2.6 Å on 115 C^α^ positions). Other structures with *Z*-scores above 8.0 show greater structural inconsistency with Tsi3 (r.m.s.d.s ranging from 3.4 to 4.0 Å on 110–119 C^α^ positions). A number of these structures were determined as the outcomes of structural genomic projects and have unknown functions. Those with known functions include the yeast Ran-binding protein MOG1P (PDB entry 1eq6; Stewart & Baker, 2000 ▶) and cyanobacterial PsbP, a zinc-binding protein (PDB entry 2xb3; Michoux *et al.*, 2010 ▶). All of the structures found by the *DALI* server show no sequence similarity to Tsi3 and can barely be overlaid on it, but all have a similar β-sandwich topography (Fig. 5 ▶ *b*). Besides these structures, the structure of Tsi3 is reminiscent of the immunoglobulin fold, which is well characterized by a β-sandwich scaffold usually comprising two four-stranded sheets. More interestingly, α-helical insertions between two β-sheet layers have been observed in some antibodies. An example is the variable domain of shark antibody IgNAR (PDB entry 2i24; Stanfield *et al.*, 2007 ▶; Fig. 5 ▶ *b*). All of these structures indicate that folds similar to Tsi3 occur widely in structural domains that mediate protein–protein interactions, and this is also the case for Tsi3.

### 3.7. Tse3–Tsi3 interactions

Tse3 and Tsi3 bind tightly to each other and form a stable binary complex in the crystal. A total of 2162 Å^2^ of solvent-accessible surface area, with 1034 Å^2^ contributed by Tse3 (6.1% of the total accessible surface) and 968 Å^2^ contributed by Tsi3 (13.6% of the total accessible surface), is buried upon complex assembly. At the interface, 20 tight hydrogen bonds (≤3.3 Å) and eight charge–charge interactions (≤6.0 Å) are formed between the two proteins; detailed information is given in Supplementary Table 2 ▶. The formation of so many noncovalent bonds and electrostatic interactions at the protein–protein interface indicates highly mutual specific recognition of both proteins. In addition, a number of hydrophobic residues, *e.g.* Val176, Leu183, Val282, Trp389 and Met391 in Tse3 as well as Pro62 and Pro130 in Tsi3, are involved in forming hydrophobic inter­actions between the two binding components. All of these analytical data prompt us to predict strong binding between Tse3 and Tsi3 and to hypothe­size that complex assembly in this case is a spontaneous process that is probably driven by both enthalpy and entropy.

**Figure 6:**
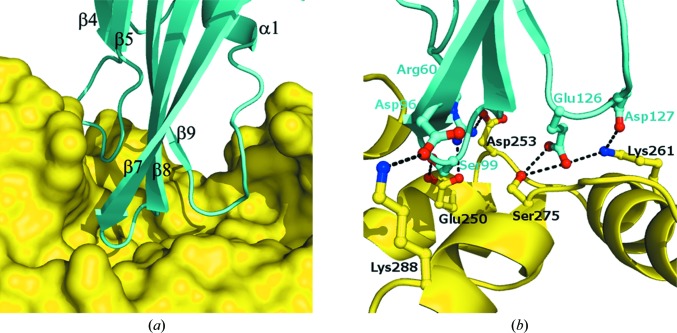
Tse3–Tsi3 interactions. (*a*) Insertion of three loops of Tsi3 (ribbon model in cyan), including two β-hairpins bridging β4–β5 and β7–β8 and a loop connecting β9 and α1, into the substrate-binding groove of Tse3 (surface representation in yellow). (*b*) The hydrogen-bond network formed between Tse3 (yellow) and Tsi3 (cyan) around the catalytic Glu residue in Tse3. The amino acids involved in hydrogen-bond formation are represented as stick models coloured by element.

The interacting residues in Tsi3 are contributed by three loops that insert deeply into the long substrate-binding groove of Tse3 (Fig. 6 ▶ *a*). The contacting segments include two β-­hairpins bridging β4–β5 and β7–β8 and the loop connecting β9 and the single α-helix, all of which are rich in charged residues. The side chains of Arg60 in the first hairpin and Asp96 and Ser99 in the second hairpin are all oriented towards Glu250, the catalytic residue in Tse3, and thus form tight hydrogen bonds with this key amino acid (Fig. 6 ▶ *b*). Coincidently, the side-chain rotamers of Arg60 and Asp96 are stabilized to a great extent by two residues carrying counter charges in the vicinity: Asp253 and Lys288 of Tse3, respectively. Consequently, a local hydrogen-bond network is formed around the catalytic centre of Tse3. Another two consecutive acidic residues, Glu126 and Asp127 in the loop following β9 in Tsi3, form hydrogen bonds to Lys261 on α18 and Ser275 on β1 in Tse3 through side-chain contacts (Fig. 6 ▶ *b*), which renders the conformation of α18 and the small β-­sheet in Tse3 more rigid upon Tsi3 binding. As a result, the adjacent Ca^2+^-binding sites (Ca1 and Ca2) harboured locally in this region are significantly stabilized, and Glu126 is directly involved in Ca^2+^ coordination as a ligating residue for Ca2.

The complex structure under study here straightforwardly reveals the structural mechanism by which Tsi3 effectively abolishes the enzymatic activity of Tse3 and neutralizes its toxicity. This may occur in three ways. Firstly, Tsi3 occludes the substrate-binding sites of Tse3 by occupying the groove space (Fig. 6 ▶ *a*). Secondly, the conformation of the active Glu residue in Tse3 is completely fixed by the hydrogen-bond network that is formed around it (Fig. 6 ▶ *b*). Lastly, the room for bound substrate is further decreased upon complex assembly owing to conformational changes of some structural elements of Tse3. For example, the space in the binding subsite +2 is insufficient to accommodate a MurNAc residue because the loop connecting α27 and α28 becomes too close to the loop between α12 and α13 (Supplementary Fig. 3 ▶a), which is probably ascribable to the formation of hydrogen bonds and electrostatic interactions between these loops and Tsi3.

### 3.8. Kinetic assay of Tse3–Tsi3 binding

**Figure 7:**
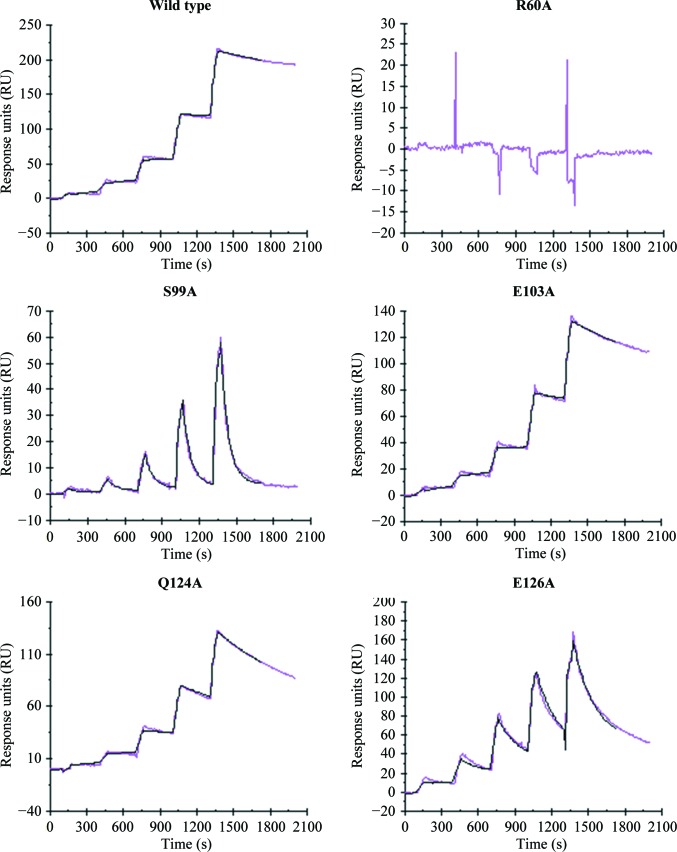
Sensorgrams from surface plasmon resonance experiments measuring the interactions of wild-type and mutant Tsi3 with Tse3. Raw data are represented by magenta curves, while the data fitted 1:1 to the Langmuir binding model are shown as black curves.

Given the extensive interaction network generated at the Tse3–Tsi3 interface, we predict high affinity between this effector–immunity pair. Consistently, in an SPR measurement performed on a Biacore 3000, wild-type Tsi3 binds immobilized Tse3 with a subnanomolar dissociation constant (*K*_d_; Table 3 ▶ and Fig. 7 ▶). This value is lower than that for Tse1–Tsi1 by an order of magnitude, but is positively correlated with the Tse3–Tsi3 interface area of 2162 Å^2^ *versus* that of 3951 Å^2^ between Tse1 and Tsi1 (Ding *et al.*, 2012 ▶). Still, such strong affinity is comparable with most antigen–antibody pairs. Consistent with our expectations, the Tsi3 mutants gave rise to *K*_d_ values that were increased from between twofold (E103A and Q124A) and several orders of magnitude (S99A and E126A), indicative of their relative contributions to the Tse3–Tsi3 interactions. Since Ser99 and Glu126 are more greatly involved in the formation of the hydrogen-bond network at the interface than Glu103 and Gln124 (Supplementary Table 1 ▶), mutations at these positions reasonably lead to relatively weaker binding. Notably, the sensorgram of R60A showed that Tsi3 completely lost its binding activity to Tse3 upon mutation at this position (Table 3 ▶ and Fig. 7 ▶), which is suggestive of the great importance of Arg60 in assembly of the Tse3–Tsi3 complex. In fact, this result agrees well with the fact that this amino acid simultaneously forms tight salt bridges to Glu250 and Asp253 of Tse3 in this structure (Fig. 6 ▶ *b* and Supplementary Table S1) and it conclusively functions as the major structural determinant in Tse3 recognition.

## 4. Discussion

The type VI secretion system of *P. aeruginosa* delivers three protein effectors, each of which strictly co-occurs with a cognate immunity protein. Among these effector–immunity pairs, structural studies on Tse3 and Tsi3 have fallen behind the other two, which have been characterized using crystallographic techniques (Benz *et al.*, 2012 ▶; Chou *et al.*, 2012 ▶; Ding *et al.*, 2012 ▶; Li *et al.*, 2012 ▶; Shang *et al.*, 2012 ▶; Zhang *et al.*, 2012 ▶; Wang *et al.*, 2012 ▶). As the first crystal structure of the Tse3–Tsi3 pair, the complex determined in our work provides a good opportunity to gain structural insights into the catalytic mechanism used by Tse3 to cleave the β-(1,4)-linkage in the cell-wall peptidoglycan of target bacteria and also the inhibition of its activity by the recognition and binding of Tsi3.

Metal ions present at or near the active site in enzymes often play catalytic roles rather than structural roles (Crow *et al.*, 2009 ▶; Li *et al.*, 2013 ▶; Luo *et al.*, 2011 ▶; Seyedarabi *et al.*, 2010 ▶). Three calcium-binding sites are surprisingly observed in the structure of Tse3, with only one ligated residue contributed by Tsi3. Our experimental data demonstrate the presence of Ca^2+^ ions at these sites. In eukaryotic proteins, calcium often serves as a key regulator in numerous biological processes, while its role in prokaryotic proteins remains elusive (Yáñez *et al.*, 2012 ▶). The single Ca^2+^ site observed in the core domain of Slt35 in *E. coli* seems to play a more structural role, as predicted from its location 20 Å away from the active site (van Asselt, Dijkstra *et al.*, 1999 ▶). In this structure, however, all three Ca^2+^ ions are bound in the substrate-binding groove of Tse3 (Figs. 3 ▶ *a* and 3 ▶ *b*), either exposed (Ca1 and Ca2) or not exposed (Ca3) to bound peptidoglycan. Their positions, in particular those of Ca1 and Ca2, are very close to the active centre, and the catalytic Glu residue even appears in the coordination shell of Ca1. This forms a striking structural feature in Tse3, as no similar sites have been observed in any other muramidases. Modelling of bound substrate by replacing *E. coli* soluble MltE by an overlaid Tse3 catalytic domain in the structure with PDB code 4hjz (Fibriansah *et al.*, 2012 ▶) shows that Ca1 seems to be completely accessible to the substrate at subsite +1, and Ca2 and Ca3 are also located near the substrate within a distance of 5 Å (Supplementary Fig. 3 ▶ *a*). Even allowing for flexible substrate binding, some atoms of the substrate are inevitably positioned at hydrogen-bonding distance from certain Ca^2+^-ligated amino acids, in particular those coordinating Ca1.

In another aspect, the richness in Glu and Asp residues, most of which are involved in Ca^2+^ coordination, notably generates a unique substrate-binding groove that has abundant negative charges (Fig. 4 ▶). This distinctive electrostatic property at the active pocket of Tse3 differs significantly from other muramidases, which contain more positive charged residues in the substrate-binding groove (van Asselt, Dijkstra *et al.*, 1999 ▶; van Asselt, Thunnissen *et al.*, 1999 ▶; Helland *et al.*, 2009 ▶; Fibriansah *et al.*, 2012 ▶). Taken together, it is reasonable to predict that the calcium ions observed in Tse3 are very likely to play more of a catalytic role than a structural role, as in the previously reported cases in pectate lyase (Seyedarabi *et al.*, 2010 ▶), adenylyl cyclase (Steegborn *et al.*, 2005 ▶) and BdbD (Crow *et al.*, 2009 ▶). They probably cause an effect in two parallel ways: (i) by direct interaction with the bound substrate *via* their ligated amino acids and/or (ii) by alteration of the electrostatic properties of the binding pocket. Since the involvement of metal ions in the catalysis of peptidoglycan degradation has never been reported, Tse3 may represent a novel class of muramidase that cleaves cell-wall peptidoglycan using an unprecedented mechanism, which would probably differ from the inverting mechanism of the goose-type lysozyme reaction, which requires a pair of catalytic acids (Hirakawa *et al.*, 2008 ▶; Helland *et al.*, 2009 ▶), or the anchimeric assistance mechanism of the lytic transglycosylase reaction, which relies on a single catalytic residue (Fibriansah *et al.*, 2012 ▶).

Structural studies of Tse1 reveal that the amidase effector adopts a typical papain-like fold and shares high structural homology with the known NlpC/P60 dl-endopeptidases; hence, a canonical mechanism of murein peptide hydrolysis is expected to be linked to Tse1 (Chou *et al.*, 2012 ▶; Ding *et al.*, 2012 ▶). Compared with amidases, glycoside hydrolases are more common enzymes, with roles in nature that include the degradation of biomass, antibacterial defence systems and cell-wall turnover (van Asselt, Dijkstra *et al.*, 1999 ▶; van Asselt, Thunnissen *et al.*, 1999 ▶; Fibriansah *et al.*, 2012 ▶). On the other hand, bacteria have generally evolved antagonistic strategies to protect themselves from cell-wall degradation. In this sense, the putative unique catalytic mechanism implemented in the glycosidic bond cleavage catalyzed by Tse3 may be significantly implicated in the competitive strategy of *P. aeruginosa* against other bacteria. One can reasonably imagine that if a peptidoglycan-cleaving effector exploiting a more general catalytic mechanism were exported from the T6SS of *P. aeruginosa*, the effector would be at risk of being inactivated by the antagonistic mechanisms existing in competing bacteria, and consequently the growth of rival bacteria cannot be effectively controlled by *P. aeruginosa*, which is certainly disadvantageous to its own growth. It follows that the bacterium may target a muramidase effector utilizing a distinctive mechanistic strategy into the periplasm of recipient Gram-negative bacteria in order to subvert their antagonistic mechanisms and thereby maximize the antibacterial efficiency. In parallel, *P. aeruginosa* utilizes a highly specific immunity protein for resisting intercellular self-intoxication. The two interrelated pathways cooperatively provide a pronounced fitness advantage for *P. aeruginosa* donor cells in the fierce niche competition.

Recently, four phylogenetically disperse families composed of peptidoglycan amidase enzymes were identified from a genomic analysis of T6SS substrates, which underscores the generality of bacteriolytic amidase effectors and cognate immunity proteins (Russell *et al.*, 2012 ▶). Likewise, the Tse3–Tsi3 muramidase effector–immunity pair is very likely not confined to *P. aeruginosa* and homologues may be identified from other Gram-negative bacteria equipped with the T6S apparatus. Further studies of these glycosidic bond-cleaving enzymes are needed in order to deepen our understanding of the underlying catalytic mechanisms, which would be of benefit for the rational design of antibacterial agents and the development of practical approaches to control pathogenic bacteria.

## Supplementary Material

PDB reference: Tse3–Tsi3 complex, 3wa5

Supplementary material file. DOI: 10.1107/S090744491301576X/dw5056sup1.pdf

## Figures and Tables

**Table 1:** Data-collection, phasing and refinement statistics of the Tse3–Tsi3 complex Values in parentheses are for the highest resolution shell.

Data collection	
Wavelength (Å)	0.9795
Space group	*P*4_3_2_1_2
Unit-cell parameters (Å, °)	*a* = *b* = 109.97, *c* = 84.95, α = β = γ = 90
Resolution range (Å)	49.18–1.90 (2.00–1.90)
Unique reflections	41573
Multiplicity	28.4 (28.8)
Completeness (%)	100.0 (100.0)
Mean *I*/σ(*I*)	28.9 (11.8)
Solvent content (%)	42.74
*R*_merge_	0.105 (0.335)
SAD phasing	
Se-atom sites	4
Overall figure of merit	0.380
Structure refinement	
*R*_work_	0.160
*R*_free_	0.202
R.m.s.d., bond lengths (Å)	0.007
R.m.s.d., bond angles (°)	1.094
Ramachandran plot	
Favoured region (%)	98.07
Allowed region (%)	1.74
Outliers (%)	0.19

**Table 2:** Metal-ion concentrations detected by ICP-AES

	Ion concentration (mg l^−1^)				
Sample	Mg^2+^	Ca^2+^	Ni^2+^	Co^2+^	Zn^2+^
Tse3 alone	0.050	0.34	<0.002	<0.002	0.003
Tse3–Tsi3	0.046	0.42	<0.002	<0.002	0.018

**Table 3:** Kinetics and affinity constants for wild-type and mutant Tsi3 binding to Tse3

Tsi3	Association rate *k*_a_ (*M* ^−1^ s^−1^)	Dissociation rate *k*_d_ (*M* ^−1^ s^−1^)	Binding affinity *K*_d_ (*M*)
Wild type	1.29 × 10^4^	3.61 × 10^−4^	2.81 × 10^−8^
R60A	Undetectable	Undetectable	—
S99A	8.99 × 10^3^	2.96 × 10^−2^	3.29 × 10^−6^
E103A	1.43 × 10^4^	6.25 × 10^−4^	4.36 × 10^−8^
Q124A	1.73 × 10^4^	1.01 × 10^−3^	5.88 × 10^−8^
E126A	1.21 × 10^4^	5.05 × 10^−3^	4.17 × 10^−7^

## Footnotes

1 Supplementary material has been deposited in the IUCr electronic archive (Reference: DW5056). Services for accessing this material are described at the back of the journal.
