# Supplementary material for: Complex structure of type VI peptidoglycan muramidase effector and a cognate immunity protein
Source: Acta Crystallogr D Biol Crystallogr. 2013 Sep 20;69(Pt 10):1889–900. doi: 10.1107/S090744491301576X (PMC3792639; doi:10.1107/S090744491301576X)
Supplement: Supplementary file 1 [file d-69-01889-sup1.pdf]

Supplementary Table S1. Bond angles of the Ca coordination spheres

**Ligand-Ca^{2+}-ligand**	**Bond angles (°)**
Ca1	
Tse3 Asn181 Oδ-Ca1-Tse3 Gln254 Oε	160.44
Tse3 Glu258 Oε1-Ca1-Wat1	152.38
Tse3 Glu258 Oε2-Ca1-Wat1	146.27
Tse3 Glu253 O-Ca1-Tse3 Gln258 Oε2	139.87
Tse3 Glu253 O-Ca1-Wat2165.45	133.59
Tse3 Gln254 Oε-Ca1-Wat2	121.36
Tse3 Asn181 Oδ-Ca1-Tse3 Glu253 O	82.70
Tse3 Glu253 O-Ca1-Tse3 Gln254 Oε	88.13
Ca2	
Tsi3 Glu126 Oε2-Ca2-Tse3 Asp262 Oδ1	102.12
Tsi3 Glu126 Oε2-Ca2-Tse3 Asp262 Oδ2	148.00
Tse3 Glu258 Oε2-Ca2-Tse3 Ser275 Oγ	125.58
Tse3 Glu258 Oε2-Ca2-Tse3 Ser275 Oγ	150.19
Tsi3 Glu126 Oε1-Ca2-Tse3 Gln280 Oε	145.84
Tse3 Asp262 Oδ1-Ca2-Tse3 Glu258 Oε2	96.01
Tsi3 Glu126 Oε2-Ca2-Tse3 Glu258 Oε2	80.52
Tse3 Asp262 Oδ2-Ca2-Tse3 Gln280 Oε	88.68
Ca3	
Tse3 Ser378 Oγ-Ca3-Tse3 Asp382 Oδ1	157.37
Tse3 Arg379 O-Ca3-Tse3 Asn384 Oδ	165.45
Tse3 Glu375 Oε1-Ca3-Wat3	148.61
Tse3 Glu375 Oε2-Ca3-Wat3	160.50
Tse3 Ser378 Oγ-Ca3-Tse3 Arg379 O	91.40
Tse3 Asp382 Oδ1-Ca3-Tse3 Asn384 Oδ	81.99
Tse3 Glu375 Oε1-Ca3- Tse3 Ser378 Oγ	71.10
Tse3 Glu375 Oε2-Ca3- Tse3 Asp382 Oδ1	84.20

Supplementary Table S2. Details of Tse3-Tsi3 interactions

**Hydrogen bonds (≤ 3.3 Å)**		
Hydrogen donor	Hydrogen accptor	Distance (Å)
Tse3: Lys171 Nζ	Tsi3: Glu129 O	2.69
Tse3: Glu258 Oε2	Tsi3: Glu126 Oε2	3.19
Tse3: Lys261 Nζ	Tsi3: Glu126 Oε1	3.12
Tse3: Lys261 Nζ	Tsi3: Asp127 Oδ1	2.78
Tse3: Ser275 Oγ	Tsi3: Ser99 O	2.87
Tse3: Ser275 N	Tsi3: Glu126 Oε1	2.91
Tse3: Ser275 Oγ	Tsi3: Glu126 Oε1	3.11
Tse3: Ser275 Oγ	Tsi3: Glu126 Oε2	3.02
Tse3: Lys288 Nζ	Tsi3: Asp96 Oδ2	3.22
Tse3: Arg379 N	Tsi3: Glu103 Oε1	2.82
Tse3: Ser386 Oγ	Tsi3: Arg60 O	2.76
Tse3: Gly388 N	Tsi3: Leu59 O	2.97
Tsi: Arg60 Nη2	Tse3: Glu250 Oε1	2.83
Tsi: Arg60 Nη1	Tse3: Asp253 Oδ2	2.85
Tsi: Ser99 Oγ	Tse3: Glu250 Oε2	2.64
Tsi3: Ser99 N	Tse3: Tyr376 O	2.97
Tsi3: Ala100 N	Tse3: Thr377 O	3.22
Tsi3: Gln124 Nε2	Tse3: Thr377 O	2.85
Tsi3: Glu126 Oε2	Tse3: Glu258 Oε2	3.19
Tsi3: Glu126 Oε1	Tse3: Asp262 Oδ1	2.97
**Ionic interactions (≤ 6.0 Å)**		
Tse3: Lys171	Tsi3: Glu129	
Tse3: Glu250	Tsi3: Arg60	
Tse3: Asp253	Tsi3: Arg60	
Tse3: Lys261	Tsi3: Glu126	
Tse3: Lys261	Tsi3: Asp127	
Tse3: Lys379	Tsi3: Asp96	
Tse3: Arg379	Tsi3: Glu103	
Tse3: Arg379	Tsi3: Asp95	

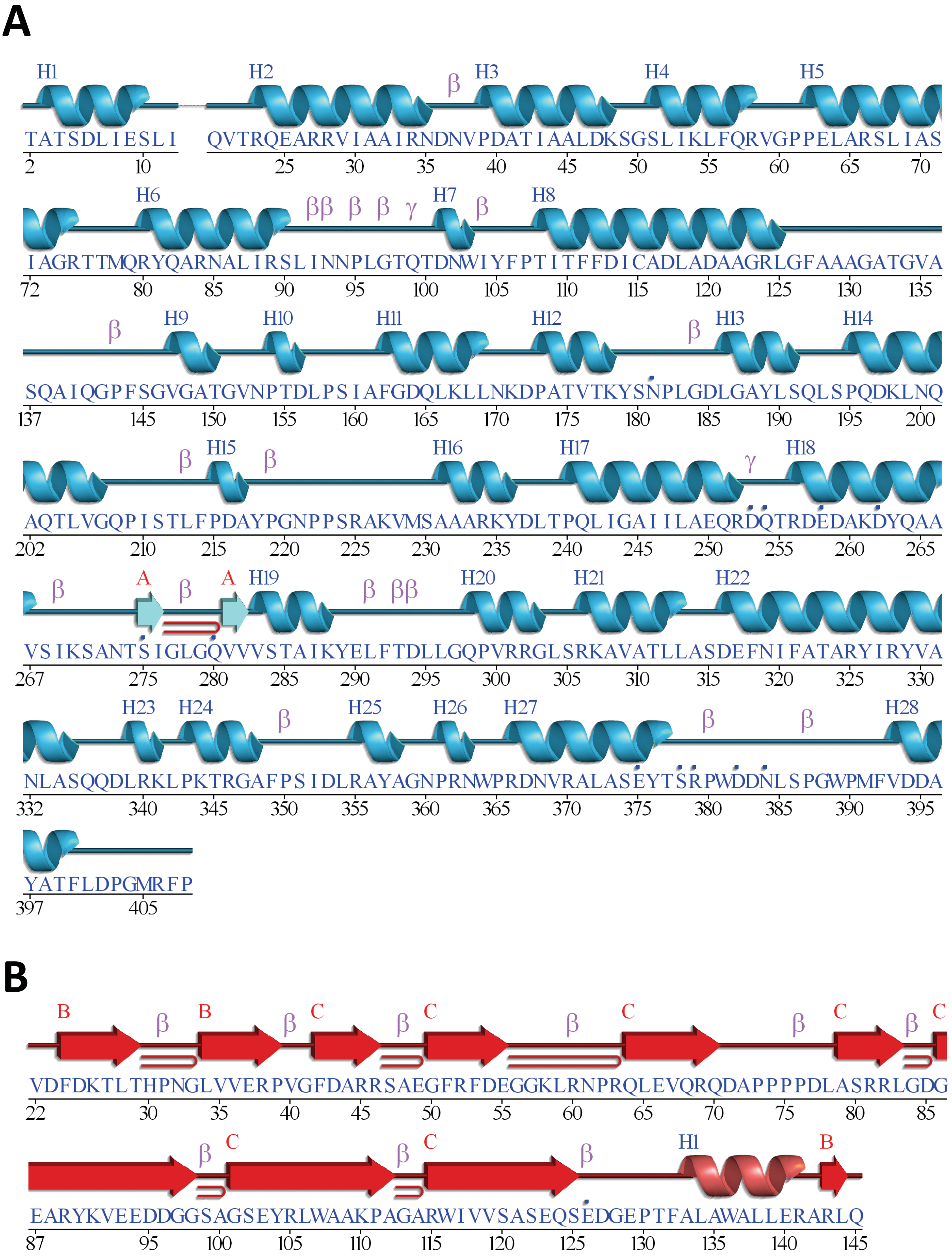

Supplementary Figure S1. Secondary structure distribution of Tse3 (A) and Tsi3 (B). α-helices and β-strands are represented by helical ribbons and schematic arrows. β-sheets are numbered as A, B and C. β-turns and γ-turns are labeled with Greek letters “β” and “γ” respectively. Residues contacting calcium ions are labeled with small blue dots.

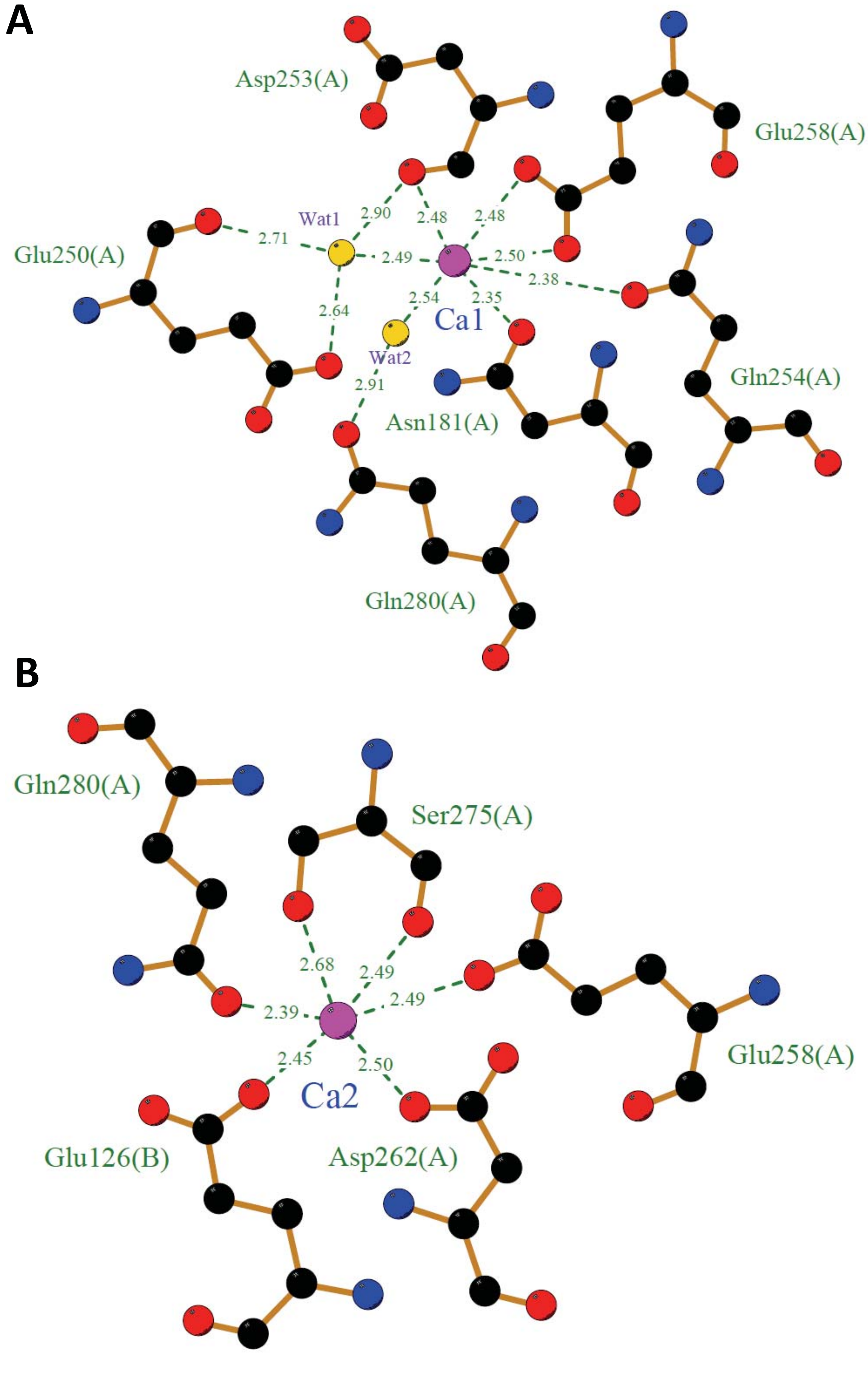

A
Asp253(A)
Glu258(A)
Glu250(A)
Wat1
2.90
2.48
2.48
2.71
2.49
2.50
2.38
2.54
2.35
2.64
Ca1
Wat2
2.91
Gln254(A)
Asn181(A)
Gln280(A)
B
Gln280(A)
Ser275(A)
2.68
2.49
2.49
2.39
2.45
2.50
Ca2
Glu258(A)
Glu126(B)
Asp262(A)

C

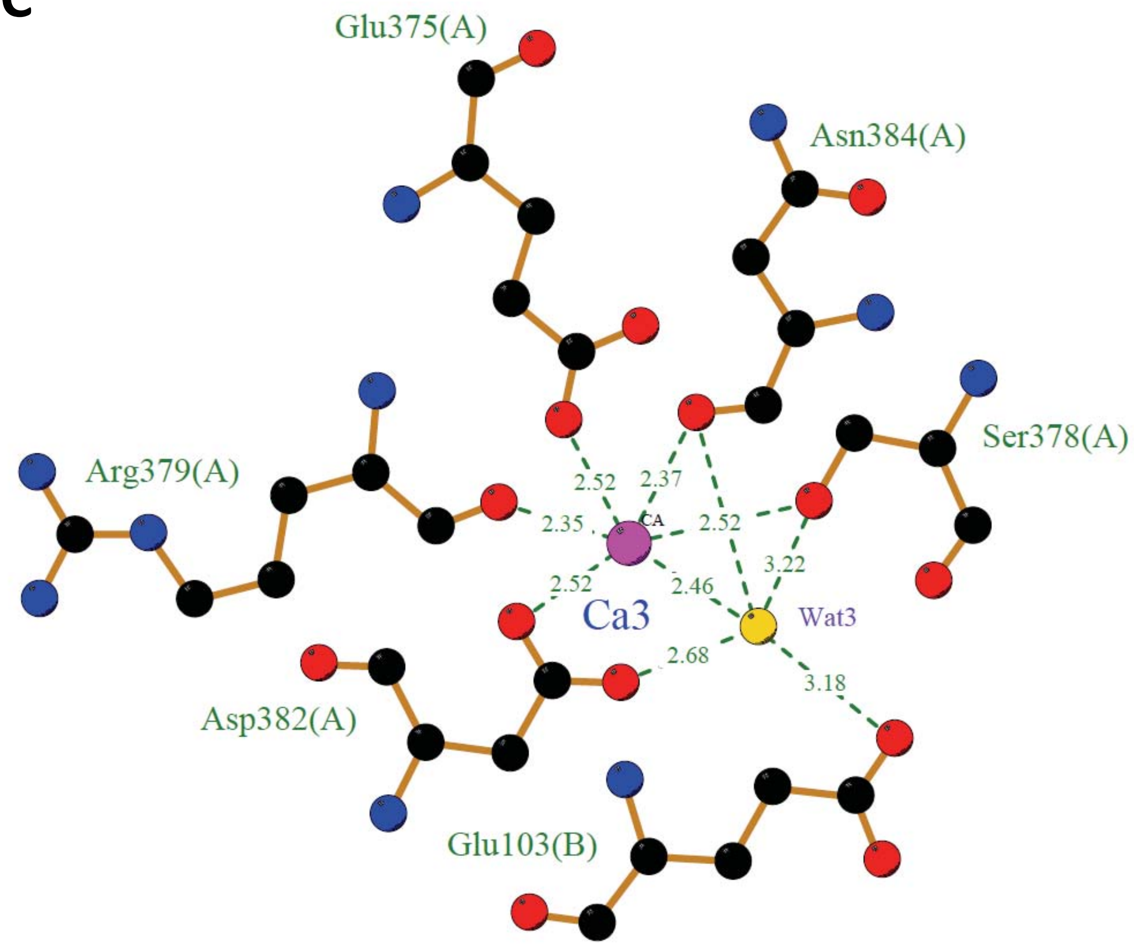

Supplementary Figure S2. Schematic diagrams showing the 3 Ca^{2+} binding sites of Ca1 (A), Ca2 (B) and Ca3 (C). The pictures were generated using LigPlot^{+} (Laskowski & Swindells, 2011).

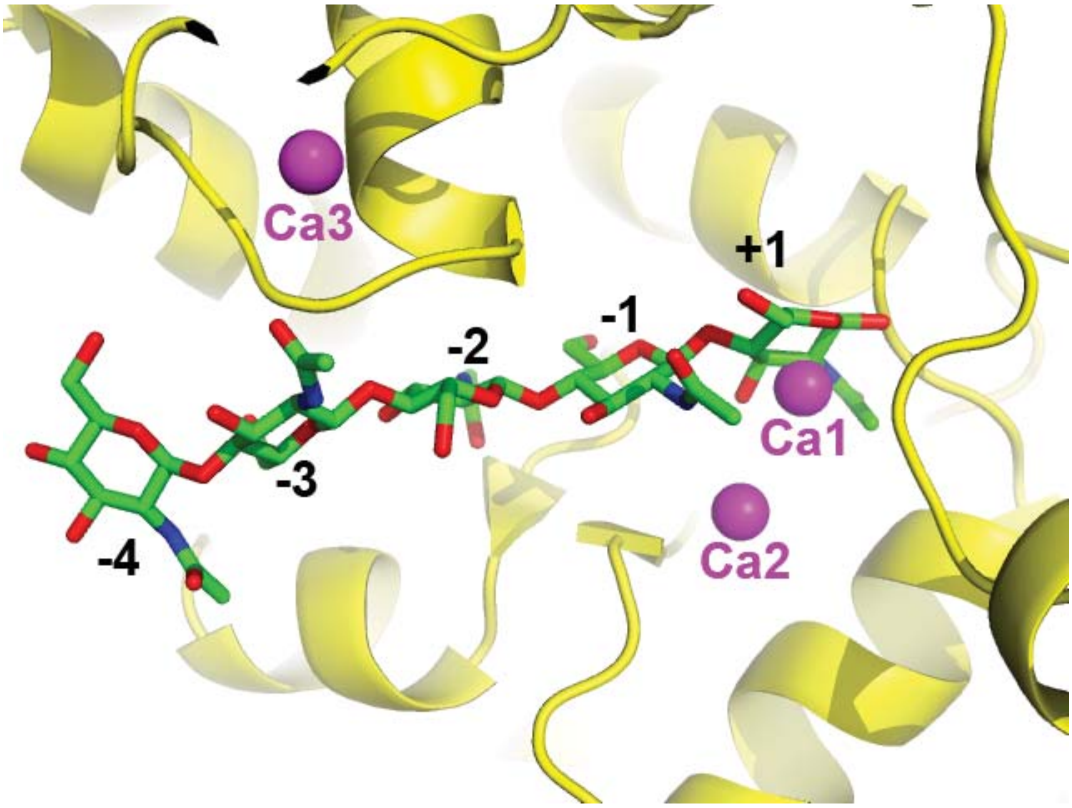

Supplementary Figure S3. Modelling of bound substrate in Tse3 by replacing *E. coli* soluble MltE with overlaid Tse3 catalytic domain in the structure with a PDB code 4HJZ (Fibriansah *et al.*, 2012). The substrate-binding subsites from -4 to +1 are labeled with reference to the position and orientation of the chitopentase present in structure 4HJZ.
